# Supplementary material for: M-CSF-stimulated myeloid cells can convert into epithelial cells to participate in re-epithelialization and hair follicle regeneration during dermal wound healing
Source: PLoS One. 2022 Jun 23;17(6):e0262060. doi: 10.1371/journal.pone.0262060 (PMC9225457; doi:10.1371/journal.pone.0262060)
Supplement: S1 Fig — Mouse splenocytes were cultured in a medium containing 5 ng/ml of M-CSF for 3 days and then suspended cells were removed by three time washing with PBS. Culture medium containing 5 ng/ml of M-CSF was changed daily. After 10 day culture, cells were examined by immunofluorescent staining and FACS analysis. (A) immunofluorescent staining with indicated antibody. DAPI (blue) was used as a nuclear counterstain. Scale bars in all images were 50 μm. (B). Adherent cells were harvested, fixed with fixation solution, stained with indicated antibody and analyzed by FACS. (PPTX) [file pone.0262060.s001.pptx]

## Slide 1
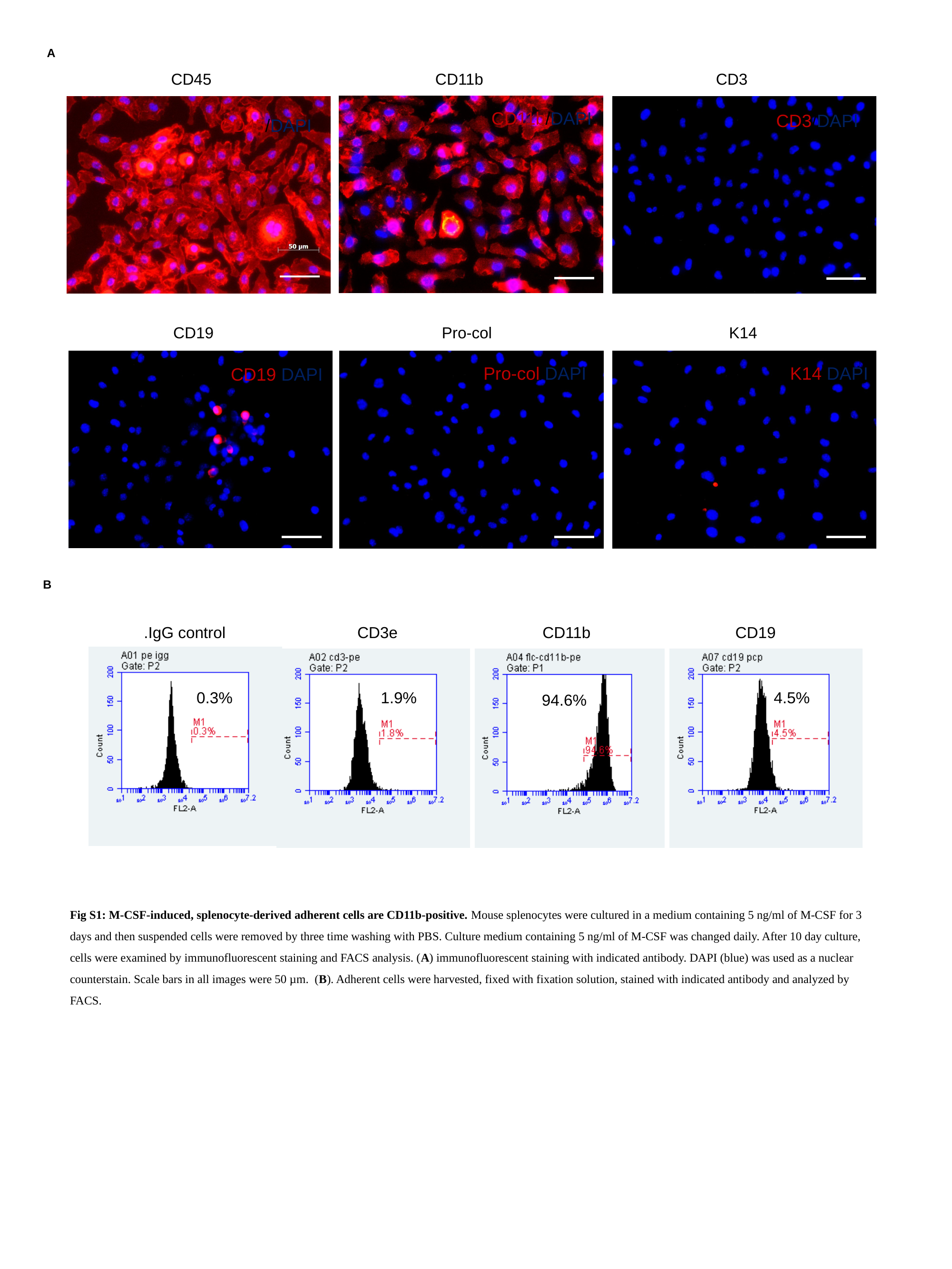

A
CD45 CD11b CD3
CD11b/DAPI
CD3/DAPI
CD45/DAPI
CD19 Pro-col K14
Pro-col/DAPI
K14/DAPI
CD19/DAPI
B
.IgG control CD3e CD11b CD19
0.3%
1.9%
4.5%
94.6%
Fig S1: M-CSF-induced, splenocyte-derived adherent cells are CD11b-positive. Mouse splenocytes were cultured in a medium containing 5 ng/ml of M-CSF for 3 days and then suspended cells were removed by three time washing with PBS. Culture medium containing 5 ng/ml of M-CSF was changed daily. After 10 day culture, cells were examined by immunofluorescent staining and FACS analysis. (A) immunofluorescent staining with indicated antibody. DAPI (blue) was used as a nuclear counterstain. Scale bars in all images were 50 µm. (B). Adherent cells were harvested, fixed with fixation solution, stained with indicated antibody and analyzed by FACS.
