# Supplementary material for: M-CSF-stimulated myeloid cells can convert into epithelial cells to participate in re-epithelialization and hair follicle regeneration during dermal wound healing
Source: PLoS One. 2022 Jun 23;17(6):e0262060. doi: 10.1371/journal.pone.0262060 (PMC9225457; doi:10.1371/journal.pone.0262060)
Supplement: S2 Fig — Dermal sections of mouse wounds on day 14 post injury were stained with CD45 antibody (green). DAPI (blue) was used as a nuclear counterstain. Scale bars in all images were 50 μm. (PPTX) [file pone.0262060.s002.pptx]

## Slide 1
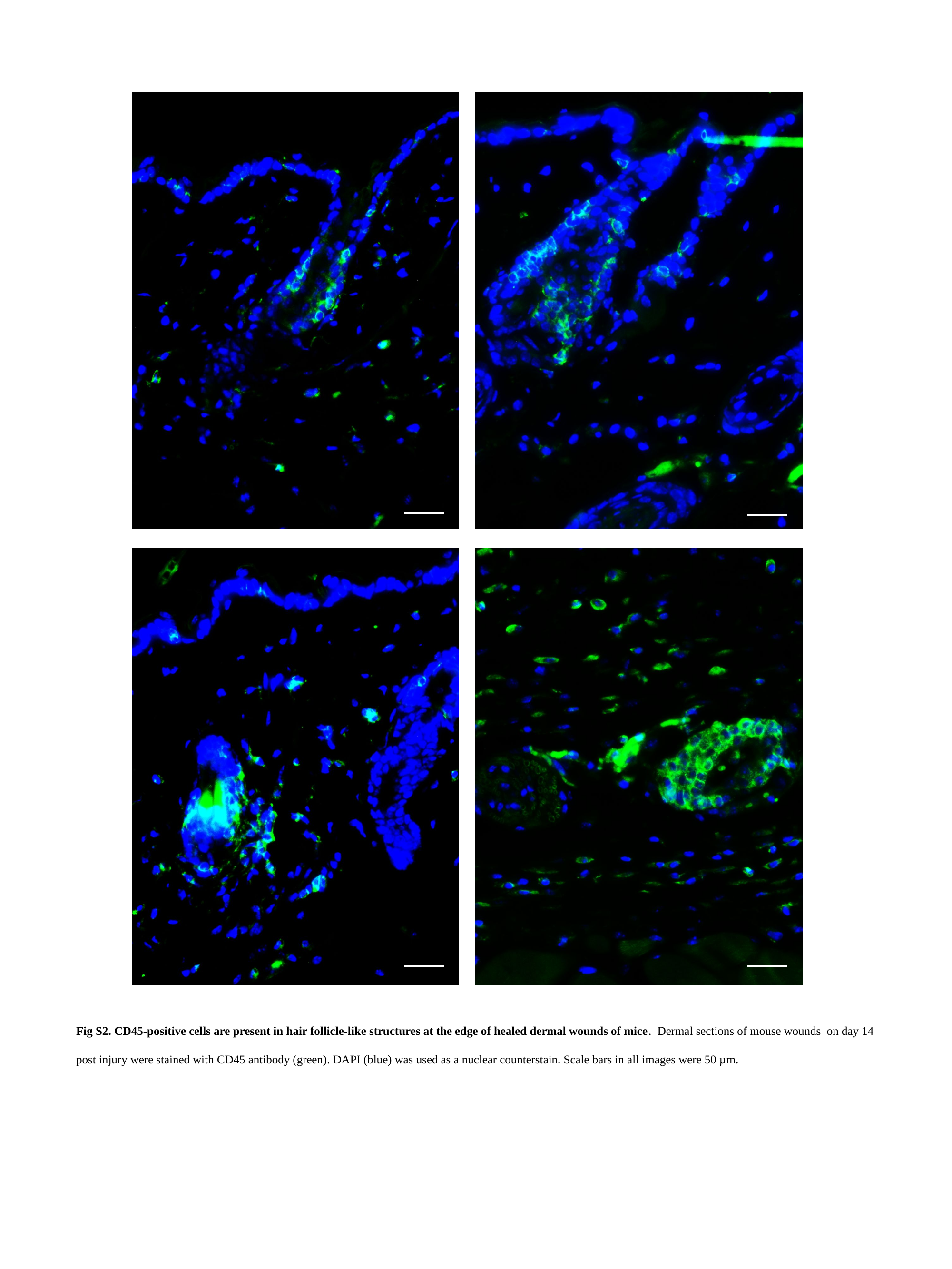

Fig S2. CD45-positive cells are present in hair follicle-like structures at the edge of healed dermal wounds of mice. Dermal sections of mouse wounds on day 14 post injury were stained with CD45 antibody (green). DAPI (blue) was used as a nuclear counterstain. Scale bars in all images were 50 µm.
