# Supplementary material for: M-CSF-stimulated myeloid cells can convert into epithelial cells to participate in re-epithelialization and hair follicle regeneration during dermal wound healing
Source: PLoS One. 2022 Jun 23;17(6):e0262060. doi: 10.1371/journal.pone.0262060 (PMC9225457; doi:10.1371/journal.pone.0262060)
Supplement: S3 Fig — Dermal sections from the wounds of mice received M-CSF-cultured myeloid cells 4 weeks post injury, were stained with CD45 antibody (red). DAPI (blue) was used as a nuclear counterstain. Scale bars in all images were 50 μm. Squares at left bottom panel were magnified and shown on the middle and right bottom panels. (PPTX) [file pone.0262060.s003.pptx]

## Slide 1
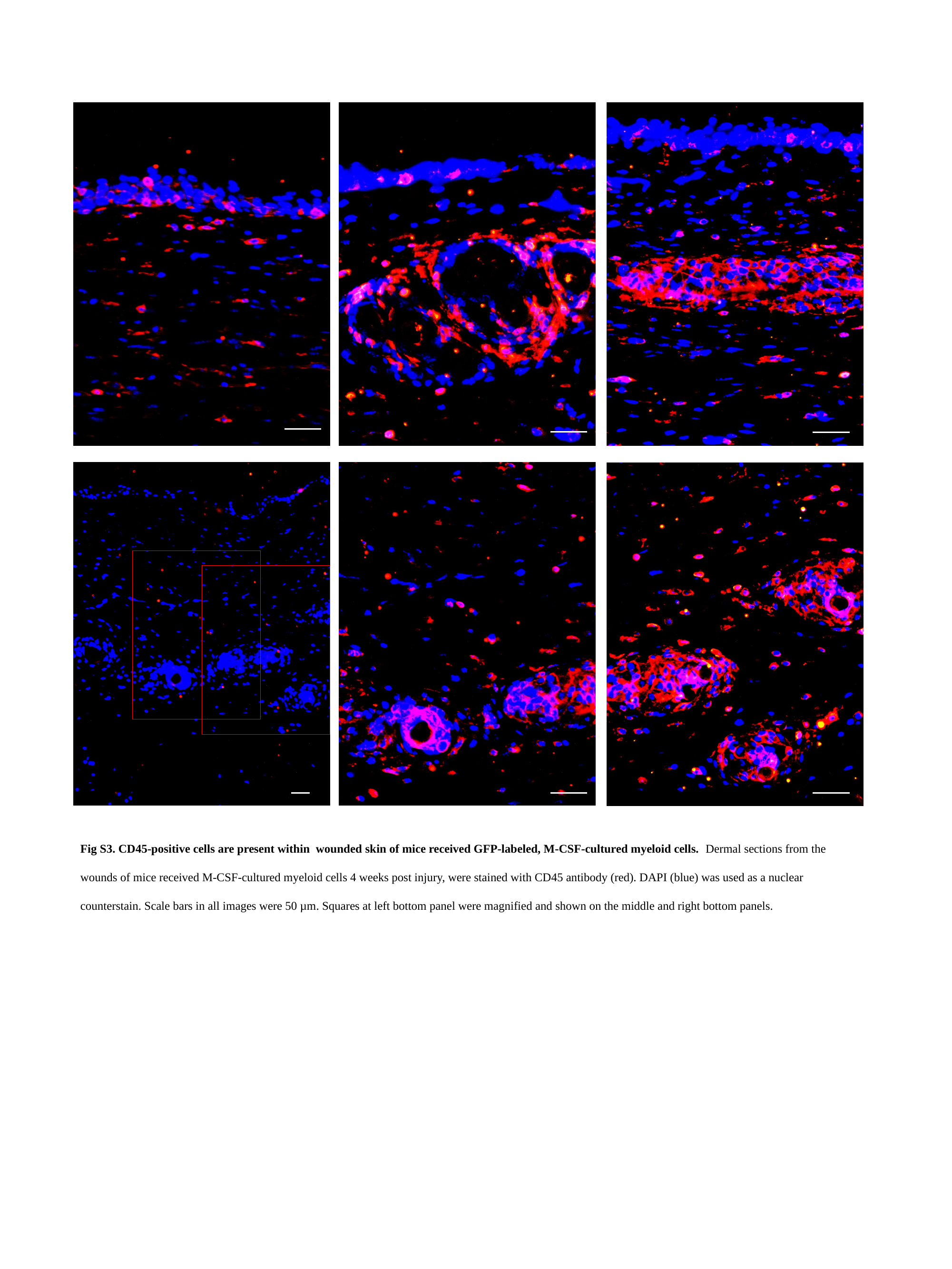

Fig S3. CD45-positive cells are present within wounded skin of mice received GFP-labeled, M-CSF-cultured myeloid cells. Dermal sections from the wounds of mice received M-CSF-cultured myeloid cells 4 weeks post injury, were stained with CD45 antibody (red). DAPI (blue) was used as a nuclear counterstain. Scale bars in all images were 50 µm. Squares at left bottom panel were magnified and shown on the middle and right bottom panels.
