# Supplementary material for: M-CSF-stimulated myeloid cells can convert into epithelial cells to participate in re-epithelialization and hair follicle regeneration during dermal wound healing
Source: PLoS One. 2022 Jun 23;17(6):e0262060. doi: 10.1371/journal.pone.0262060 (PMC9225457; doi:10.1371/journal.pone.0262060)
Supplement: S4 Fig — Mice received dermal injection of one million GFP-positive, M-CSF-cultured myeloid cells were created excisional dermal wounds by punch biopsy. Mice were euthanized after 4 weeks and skin was collected and immunofluorescent stained for detection of GFP (green). DAPI (blue) was used as a nuclear counterstain. Scale bars in all images were 50 μm. (PPTX) [file pone.0262060.s004.pptx]

## Slide 1
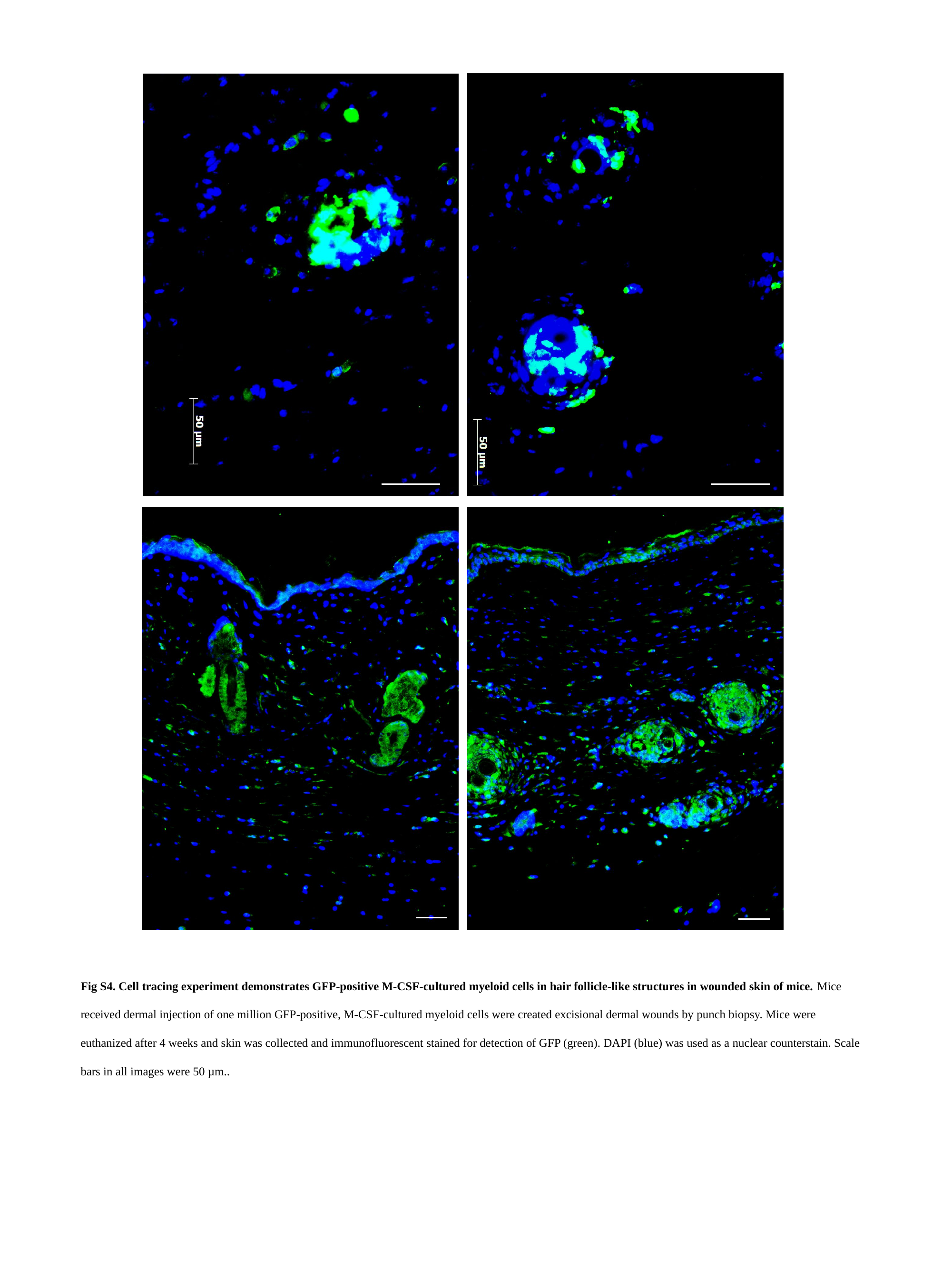

Fig S4. Cell tracing experiment demonstrates GFP-positive M-CSF-cultured myeloid cells in hair follicle-like structures in wounded skin of mice. Mice received dermal injection of one million GFP-positive, M-CSF-cultured myeloid cells were created excisional dermal wounds by punch biopsy. Mice were euthanized after 4 weeks and skin was collected and immunofluorescent stained for detection of GFP (green). DAPI (blue) was used as a nuclear counterstain. Scale bars in all images were 50 µm..
