# Supplementary material for: M-CSF-stimulated myeloid cells can convert into epithelial cells to participate in re-epithelialization and hair follicle regeneration during dermal wound healing
Source: PLoS One. 2022 Jun 23;17(6):e0262060. doi: 10.1371/journal.pone.0262060 (PMC9225457; doi:10.1371/journal.pone.0262060)
Supplement: S1 Table — (DOCX) [file pone.0262060.s005.docx]

| **Antibody name** | **host** | **use** | **concentration** | **Source** | **Cat#** |
| --- | --- | --- | --- | --- | --- |
| CD45 | Rat | IF | 1:500 | eBiosci | 14-0451-81 |
| CD11b | Rat | IF (culture cells) | 1:500 | eBioSci | 14-0112-82 |
| CD11b | Rabbit | IF (tissue) | 1:1000) | Abcam | Ab133357 |
| GFP | Rabbit | IF | 1:1000 | Abcam | Ab6556 |
| Keratin-14 | Mouse | IF | 1:1000 | Abcam |  |
| Anti-mouse IgG Alexa Fluor 568 | goat | IF | 1:1000 | Invitrogen | A11004 |
| Anti-mouse IgG Alexa Fluor 488 | Goat | IF | 1:1000 | Invitrogen | A11029 |
| Anti-rat IgG-Alexa Fluor 488 | goat | IF | 1:1000 | Invitrogen | A11006 |
| Anti-rat IgG-Rhodamine red | goat | IF | 1:750 | Jackson ImmunoRes. Lab. | 112-295-003 |
| Anti-rabbit IgG Rhodamine red | goat | IF | 1:750 | Jackson ImmunoRes. Lab. | 111-295-003 |
| Anti-rabbit IgG Alexa Fluor 488 | Goat | IF | 1:1000 | Invitrogen | A11008 |
| CD3e-PE | Rat | FCM | 1:100 | eBiosci | 12-0031-85 |
| CD19-PCP | Rat | FCM | 1:100 | eBiosci | 50-0193-82 |
| CD11b-PE | Rat | FCM | 1:100 | eBiosci | 12-0112-83 |

IF: immunofluorescence; FCM: flow cytometry

**Table S1:** Source and concentration of primary and secondary antibodies
